# Highly Efficient Water Harvesting with Optimized Solar Thermal Membrane Distillation Device

**DOI:** 10.1002/gch2.201800001

**Published:** 2018-05-24

**Authors:** Guobin Xue, Qian Chen, Shizhe Lin, Jiangjiang Duan, Peihua Yang, Kang Liu, Jia Li, Jun Zhou

**Affiliations:** ^1^ Wuhan National Laboratory for Optoelectronics and College of Optoelectronic Science and Engineering Huazhong University of Science and Technology Wuhan 430074 China

**Keywords:** membrane distillation, solar thermal, water harvesting

## Abstract

Water distillation with solar thermal technology could be one of the most promising way to address the global freshwater scarcity, with its low cost and minimum environmental impacts. However, the low liquid water productivity, which is caused by the heat loss and inadequate heat utilization in solar‐thermal conversion process, hinders its practical application. Here, a compact solar‐thermal membrane distillation system with three structure features: highly localized solar‐thermal heating, effective cooling strategy, and recycling the latent heat, is proposed. The steam generation rate is 0.98 kg m^−2^ h^−1^ under solar illumination of 1 kW m^−2^ in the open system, while the liquid water productivity could be 1.02 kg m^−2^ h^−1^ with the solar efficiency up to 72% with a two‐level device. The outdoor experiments show a water productivity of 3.67 kg m^−2^ with salt rejection over 99.75% in one cloudy day. These results demonstrate an easy and high‐efficiency way for water distillation, especially suitable for household solar water purification.

## 1 Introduction

Freshwater scarcity is one of the most pervasive problems throughout the world, threatening two‐thirds of the world's population.^1^ Millions of people die every year from water‐borne diseases.^2^ Energy from sun to earth is as high as 3 × 10^24^ Joules year^−1^, and converting this huge solar energy to heat for water distillation can potentially solve this society's problem with minimum environmental impacts.^3^ Considering the sun flux of 1 kW m^−2^ and water evaporation enthalpy of 2454 kJ kg^−1^, the steam productivity could be 1.46 kg m^−2^ h^−1^ with no optical concentration, which would meet the basic household drinking water demand.

Recently, solar steam generation was widely studied with a structure of solar thermal localization.^4^ Comparing to conventional technologies which rely on costly and cumbersome optical concentration systems to heat a bulk liquid, this structure reduces the heat loss to the bulk water by localizing the solar heat at the evaporation surface.[[qv: 3b,5]] Most related studies have been focused on high efficiency solar absorption materials, such as plasmonic and carbon‐based materials, in which quite high solar‐thermal conversion efficiency was achieved.[[qv: 3e,f,6]] However, in these studies, only steam was generated and the liquid water was hard to harvest.[[qv: 3c,7]] That is because the steam needs to be condensed on a transparent cover, which has two disadvantages. First, condensed water together with the transparent cover hinders the incident sunlight. Second, the cover will be heated with the vapor and sun, further hindering the condensation process. And especially in the hot season, condensing the vapor with the cover is rather slow as the high outdoor temperature goes against the latent heat releasing. As a result, although high solar‐thermal conversion efficiency is achieved, the liquid water harvesting which is critical for practical application is always inefficient.

Membrane distillation (MD) is a hybrid thermal/membrane technology, which can operate at lower temperatures than conventional thermal distillation (i.e., boiling) and lower pressures than reverse osmosis (RO), and maintains the highest NaCl rejection.^8^ For most MD process, heated saline water (feed) and cold purified water (distillate) are separated by a microporous hydrophobic membrane. The fluid exchange is prevented by the hydrophobic membrane and the mass transfer takes place in vapor phase driven by the vapor‐pressure gradient. As the vapor pressure of hot feed is larger, the vapor will transfer from the hot feed to the cold distillate. Since water owns a huge evaporation latent heat, the vapor will give off a lot of latent heat when condensing at the cold side and reduce the temperature difference across the membrane.^9^ As a result of this “temperature polarization” effect, the net driving force to the mass transfer decreases and the efficiency of the distillation process drastically falls down.

Here, we report a new approach of solar MD system and corresponding material structure that harvests liquid water with high solar efficiency and salt rejection. In the water‐harvesting process, the vapor was forced flow downward and condensed with cold bulk water. The high temperature difference between the vapor and the collector ensured the enough drive force for this flow. The latent heat was recycled to optimize the heat utilization.^10^ The porous hydrophobic membrane in this MD system ensured the high salt rejection. This structure had three features, highly localized solar‐thermal heating, effectively condensing the vapor with cold bulk water, and recycling latent heat, which contributed to a high solar efficiency and water productivity. The liquid water productivity was 1.02 kg m^−2^ h^−1^ with a two‐level collector device, achieving a solar efficiency as high as 72% under solar illumination of 1 kW m^−2^. Under a natural solar flux, this device could collect 3.67 kg m^−2^ water 1 d. This highly compact solar‐thermal membrane distillation apparatus with simple structure and high efficiency was easily assembled, especially suitable for personal operation in practical application.

## 2 Results and Discussion

**Figure 1:**
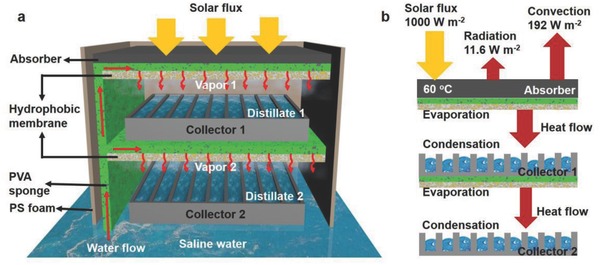
Schematic of high‐efficient solar‐thermal membrane distillation system. a) The cross‐sectional view to exhibit every component of the system and the water transfer process. b) Energy balance and heat transfer diagram for an absorber (assuming reaching 60 °C) with thermal emittance of 5% under the solar flux of 1000 W m^−2^.

To efficiently harvest water with the ambient solar flux, heat loss should be reduced and the heat utilization should be optimized.^11^ **Figure** **1**a showed the schematic structure of the high efficient liquid water‐harvesting system with solar energy. The solar energy is the only power source of this system. The absorber was heated up with solar flux and formed a hot spot. Bulk saline water was wicked automatically by polyvinyl alcohol (PVA) sponge to the hot spot and generated vapor. Then the vapor was forced downward to go through the hydrophobic membrane and condensed on the collector. The hydrophobic membrane here was a layer of poly(vinylidene fluoride‐*co*‐hexafluoropropylene) (PVDF‐HFP) nanofibers network on the back side of PVA. In this system, capillary force replaced the complex flow driving units in conventional MD system and no external driving energy was needed. Due to the low thermal conductivity of PVA sponge, small amount of heat conducted to the bulk water (Figure S1, Supporting Information).^4^ The condenser was not contact with the membrane to prevent heat transfer to bulk water by heat conduction. Thus, solar energy was highly localized at the hot internal region and the vapor will be heated to a rather high temperature. Comparing to the high temperature vapor, the bulk water was relatively “cold” and was suitable to condense the vapor. The collector has a height of about 1 cm and the fins have a thickness about 1 mm (Figure S2, Supporting Information). It should be noted that the collector also worked as condenser here, with many fins to increase the thermal conduction capability and transfer the heat to the cold bulk water. Since the latent heat released to the condenser, the temperature difference across the membrane is kept at a relatively high value, which effectively forced the vapor flow downward. “Temperature polarization” was avoided as the heat flow from the sun offset the latent heat of the evaporated water and the latent heat released to cold bulk saline water.

Figure 1b shows the energy transfer process assuming that the absorber reached 60 °C under a solar flux of 1000 W m^−2^. The main heat loss *Q*_loss_ can be calculated as follows

(1) $$Q_{\mathrm{loss}}=A(1-\alpha )Q_{s}+A\varepsilon \sigma \left(T^{4}\;\;-\;\;T_{a}{}^{4}\right)+Ah\left(T-T_{a}\right)+Q_{\mathrm{water}}$$

where *A* is the total area of the absorber, *Q*_s_ the power of solar irradiation, α the solar absorptance, ε the emittance of the absorber, σ the Stefan–Boltzmann constant, *h* the convection heat transfer coefficient (natural convection heat transfer coefficient is about 10 W m^−2^ K), *T* the temperature of the absorber, *T*_a_ the ambient temperature, and *Q*_water_ the heat flux to underlying water. The heat radiation loss can be very little for an absorber with a low emittance. The convection loss can be reduced with a transparent cover to weaken convection.^12^ The side areas of the device were packaged with low thermal conductivity materials, polystyrene (PS) foam, which assured the heat being enclosed in the system. Thus, the solar efficiency could be rather high since the energy loss is main because of the slight heat radiation and convection on the surface of the absorber.^13^ Moreover, two‐level collectors were designed for utilizing the latent heat. As shown in the lower part of Figure 1a, the first level collector worked as a thermal source, which heated the next level PVA sponge to generate vapor, further enhancing the water productivity. It should be noted that in this passive system the water productivity is subject to limited condensation process. For example, thermal resistance from the noncondensable gases cannot be reduced. However, considering the easily running and economic feasibility, this system is promising in isolated and impoverished areas.

**Figure 2:**
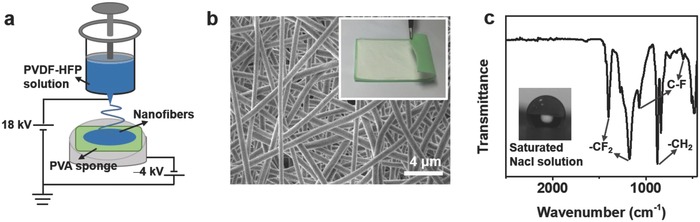
The hydrophobic membrane. a) The schematic of electrospinning PVDF‐HFP nanofibers membrane directly on PVA sponge. b) The SEM image of the PVDF‐HFP nanofibers membrane. The inset showed the photograph of PVDF‐HFP/PVA sponge. c) The FTIR spectra of PVDF‐HFP membrane. The inset showed the contact angle of PVDF‐HFP membrane with saturated NaCl solution.

In MD system, the membrane is the key component part, which should keep the hydrophobic ability even in strong brine. Here, hydrophobic PVDF‐HFP nanofibers were electrospun directly on the surface of PVA sponge surface (**Figure** **2**a). The thickness of the PVA sponge is about 2 mm. To assure the nanofibers depositing on the PVA sponge surface, an electrode with the same area was adhered just below the PVA sponge and a negative voltage of 4 kV was applied in the electrospinning process. As shown in the inset of Figure 2b, a uniform white membrane (with a thickness about 80 µm) was electrospun on one side of green PVA sponge. The nanofibers have an average diameter about 600 nm and arrange disorderly to form a dense network (Figure 2b). Figure 2c showed the Fourier transform infrared (FTIR) spectra of the PVDF‐HFP copolymer nanofibers. The peaks centered at 480 and 840 cm^−1^ are attributed to β and γ phases, respectively.^14^ Due to the rich fluorine‐containing groups, such as —CF_2_ and C—F, the membrane could maintain its high hydrophobic ability in strong brine, with a contact angle of 123° for saturated NaCl solution (insets of Figure 2c). It should be noted that MD is must adopted here since without the hydrophobic membrane salt water from the sponge is easy to pollute the distill below.^15^

**Figure 3:**
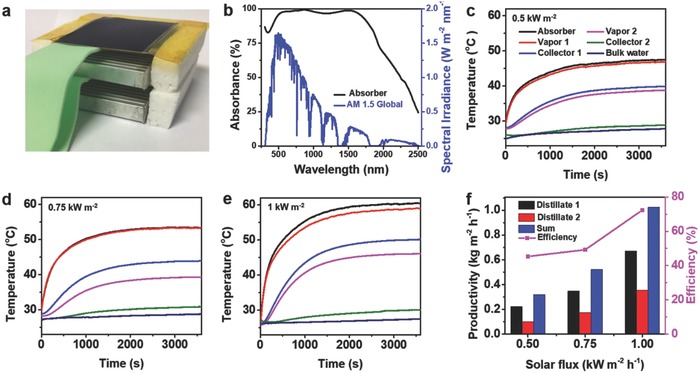
Water harvesting with the solar‐thermal membrane distillation system. a) The cross‐sectional view of the device. b) The absorption spectrum of the absorber in the wavelength ranging from 380 to 2500 nm, weighted by standard AM 1.5 G solar spectrum. The temperature of vapor and collector under c) 0.5, d) 0.75, and e) 1 kW m^−2^ solar flux in this two‐level collector device. f) The corresponding water productivity and solar efficiency of the two‐level collector device under different solar fluxes.

We then assembled a device to test the water‐harvesting ability (**Figure** **3**a). The absorber here will transfer the solar energy to heat for running this MD system. We used a commercial spectrally selective absorber (Bluetec, Germen) here, which consisted of a commercially available cermet‐coated aluminum alloy substrate. Figure 3b shows the absorption spectra in the solar spectrum range from 300 to 2500 nm. Comparing to convectional black materials, such as carbon‐based materials, this spectrally selective absorber had a high solar absorption and emitted very little radiative heat.^12^, ^16^ The solar absorbance was ≈95% and the thermal emittance was ≈5%. In practical application, the solar intensity always tends to fluctuate with the changeable weather. So we tested the temperature of the vapor and condenser (the collector) under different solar fluxes, as shown in Figure 3c–e. The steady temperature of vapor and the temperature difference between the vapor and collector both increased with the increasing solar flux. The temperature of bulk saline water stayed at a low value. The temperature of vapor increased quickly, which could be attributed to the highly localized solar‐thermal heating. The quick increase of vapor temperature would ensure the efficiency of water collection in cloudy days. And the higher the incident light flux was, the higher the temperature of vapor and the temperature difference between vapor and collector. As shown in Figure 3e, the first level vapor is about 59 °C (with a vapor pressure about 19 kPa), and the second level collector is about 30 °C (with a vapor pressure about 4.2 kPa). This high temperature difference could assure enough drive force for the vapor flowing downward. Comparing to the device with one‐level collector (Figure S3, Supporting Information), the steady absorber temperature here was higher, which would benefit the optimization of heat utilization.

The solar efficiency (η) was calculated as

(2) $$\eta =m\;h_{w}/\left(AQ_{s}\right)$$

where *m* is the water productivity in the condenser or mass flux of the vapor, and *h*_w_ is water evaporation enthalpy. Figure 3f shows the water productivity and solar efficiency at different solar fluxes. The water productivity increased with the enhanced solar flux. The productivity of the second level collector was about half of the first level collector. Under a solar flux of 1 kW m^−2^, the total water productivity was about 1.02 kg m^−2^ h^−1^, achieving a solar efficiency about 72%, with an obvious enhancement comparing to one‐level system (0.727 kg m^−2^ h^−1^, 51%, Figure S3d, Supporting Information). It should be noted here that the liquid water productivity of the two‐level device is also high than the vapor flux in open system (0.98 kg m^−2^ h^−1^, Figure S1, Supporting Information), which confirm the importance of latent heat recovery. Salt rejection was defined as the percent reduction in salinity between the distillate and the feed, calculated using the following equation^17^

(3) $$\mathrm{Salt}\;\mathrm{rejection}\;\left(\%\right)=\left(1-\sigma_{d}/\sigma_{f}\right)\times 100\%$$

where σ_d_ is the conductivity of the harvested water, and σ_f_ is the conductivity of the bulk feed. The ion concentration of harvested water was decreased to a fairly low level, about 25 mg L^−1^ (42 µS cm^−1^), which is much lower than the salinity levels defined by World Health Organization for drinking water (103 mg L^−1^). Considering the conductivity of the feed saline water (0.6 m NaCl solution) was 52 mS cm^−1^, we achieved a salt rejection of 99.9% here.

**Figure 4:**
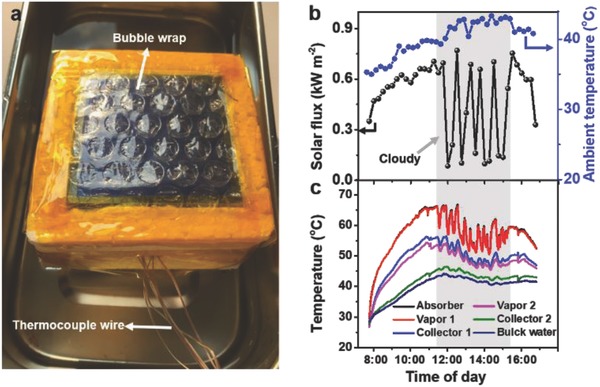
Water harvesting outdoors. a) Image of a water‐harvesting prototype outdoors. b) Solar flux and temperature outdoors from 7:45 a.m. to 4:45 p.m. on 26 July, 2017, at the campus of Huazhong University of Science and Technology. The fluctuation of the solar flux was due to the presence of clouds. c) The corresponding temperature of vapor and collector.

Outdoor experiment was also conducted to demonstrate the viability of this two‐level collector prototype. One bubble wrap covering the absorber was used to decrease heat convection to air (**Figure** **4**a).^12^ In practical test, all bulk saline water were hidden with PS foam to avoid being heated by sunlight. Figure 4b shows the ambient air temperature and solar flux on a sunny day, which varied dramatically with roaming cloud. The corresponding temperature of vapor and collector under different solar fluxes is shown in Figure 4c. Due to the highly localized solar‐thermal heating, the temperature response of vapor to the solar flux was quick, which would contribute to establish the high temperature difference for vapor condensation. After measurement from 7:45 a.m. to 4:45 p.m. on 26 July 2017 in Wuhan, the total water productivity was about 3.67 kg m^−2^ (2.6 and 1.07 kg m^−2^ for the first and second level collector, respectively), denoting that this prototype would provide enough water for one adult's daily needs with one square meter. After 1 d of operation, no salt was rejected from the PVA sponge and the PVDF‐HFP membrane maintained its hydrophobic ability, which proves the stability of this system. The salt accumulated in the evaporators may diffuse back into the saltwater basin due to concentration gradient and gravity.^18^ In practical application, the PVA sponge may also be used as a filter to absorb surfactant in seawater which may fuel the hydrophobic membrane. On the other hand, since our system is easy to assemble and disassemble, the PVA sponge can be conveniently cleaned or replaced. The conductivity of the produced water was 128.8 and 132.4 µS cm^−1^ for the first and second level collector, respectively, denoting a salt rejection over 99.75%. This impressive water harvesting confirms the role of thermal localization and latent heat recycling. Further improvement of the water harvesting can be expected if we optimize the heat and mass transfer process by increasing the collector levels with optimized characteristic distance of every evaporation/condensation level. The condensation process can be improved with a more hydrophobic collector.^19^ Furthermore, this system has the characteristics of using low cost, easily obtained material, convenient assembly and disassembly, being capable of replicating and popularizing, and can be further scaled up easily after stringing several devices together at will.

## 3 Conclusion

In summary, we prepared a compact and efficient solar‐thermal membrane distillation system. Highly localized solar‐thermal heating was achieved by the spectrally selective absorbing and thermal insulating to bulk water for generating high‐temperature vapor. The high‐temperature vapor was effectively cooled and condensed with the relatively cold bulk water. By latent heat recycle, the water productivity and solar efficiency can be further enhanced. A two‐level device achieves water productivity as high as 1.02 kg m^−2^ h^−1^ with a solar efficiency of 72% under 1 sun illumination. The high salt rejection is ensured with the hydrophobic membrane. This work, in principle, highlights a practical guideline for further development of personal water harvesting with solar thermal membrane distillation system on the basis of heat localization, condensing with cold bulk water, and latent heat recycling strategies.

## Conflict of Interest

The authors declare no conflict of interest.

## Supporting information

Supplementary
