# Supplementary material for: Highly Efficient Water Harvesting with Optimized Solar Thermal Membrane Distillation Device
Source: Glob Chall. 2018 May 24;2(5-6):1800001. doi: 10.1002/gch2.201800001 (PMC6607162; doi:10.1002/gch2.201800001)
Supplement: Supplementary file 1 — Supplementary [file GCH2-2-1800001-s001.pdf]

Copyright WILEY-VCH Verlag GmbH & Co. KGaA, 69469 Weinheim, Germany, 2018.

Global Challenges
Open Access

Supporting Information

for *Global Challenges*, DOI: 10.1002/gch2.201800001

Highly Efficient Water Harvesting with Optimized Solar Thermal Membrane Distillation Device

*Guobin Xue, Qian Chen, Shizhe Lin, Jiangjiang Duan, Peihua Yang, Kang Liu, Jia Li, and Jun Zhou**

Supporting Information

Highly efficient water harvesting with optimized solar thermal membrane distillation device

*Guobin Xue†, Qian Chen†, Shizhe Lin†, Jiangjiang Duan, Peihua Yang, Kang Liu, Jia Li, Jun Zhou**

Wuhan National Laboratory for Optoelectronics and College of Optoelectronic Science and Engineering, Huazhong University of Science and Technology, Wuhan, 430074, China.

E-mail: junzhou@hust.edu.cn

† These authors contributed equally to this work.

Keywords: water harvesting, solar thermal, membrane distillation

Table of content:

1. Experimental details

2. Supplementary Figures

1. Experimental details

Synthesis of the PVDF-HFP nanofibers membrane. The hydrophobic nanofiber network was directly electro-spun on the surface of PVA sponge. In briefly, Poly(vinyluidene fluoride-co-hexafluoropropylene) (PVDF-HFP),

N,N-dimethylformamide (DMF) and acetone with a mass ratio of 1:3.6:1.2 were blended to form a transparent solution after magnetically stirring for several hours. This solution was then ejected from the stainless steel capillary with a feeding rate of 1.0 mL h^{-1}. The distance between the capillary and collector were 15 cm. To assure the uniform distribution of the nanofiber network, a positive voltage of 18 kV and a negative voltage of 4 kV were applied on the capillary and collector respectively.

Characterizations. The solar light was supplied by a solar simulator (Newport). The water productivity at the condenser was measured with a high accuracy balance (Mettler Toledo, ME204E) after the system working for one hour. The temperature was measured by thermocouples and recorded by a data logger (TC-08). The salt concentration test was performed with a conductivity meter (Mettler Toledo, S230 SevenCompact™). All indoor tests were conducted with sodium chloride (NaCl) solution of 0.6 M in ambient temperature of 28 ^{o}C and relative humidity of 50%. The pressure drop across the PVDF-FHP membrane was measured with a pressure transmitter (DP102, Sike instruments). The thickness of the PVDF-HFP membrane was measured with micrometer.

2. Supplementary Figures

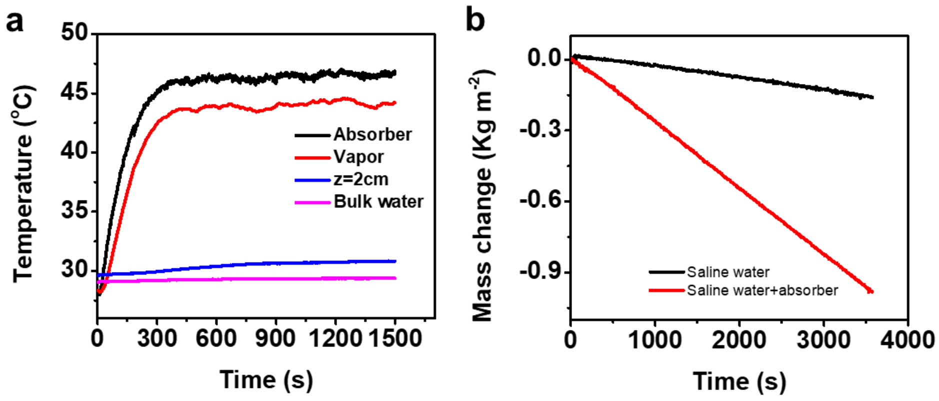

Figure S1. Heat localization. (a) The temperature of steam and bulk water after solar irradiation under 1 kW m^{-2} solar irradiation in open air. The temperature of evaporation surface and absorber increase quickly after a solar irradiation, and reach a steady state (about 43 ^{o}C) in about 7 min. The temperature of PVA sponge at 2 cm away from the evaporation surface and the bulk water did not significant change. All this demonstrates that heat did not transfer to the bulk water and was localized at the evaporation surface. (b) The evaporation induced mass change of water for water with heat localization comparing with pure saline water. The water evaporation rate with thermal-localization structure is 0.98 kg m^{-2} h^{-1}, about 6 times comparing to pure NaCl solution (0.16 kg m^{-2} h^{-1}).

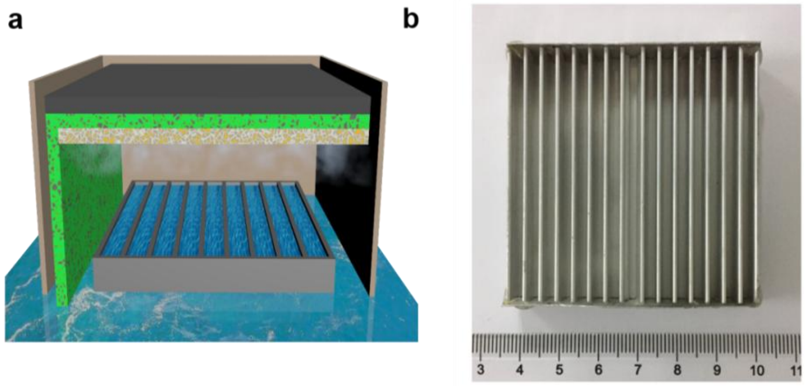

Figure S2. (a) Schematic picture of the one level collector device. (b) The photo of an aluminium collector. The size of collector is the same with the absorber, 6× 6 cm^2. The thickness of fins and walls of the collector is about 1 mm.

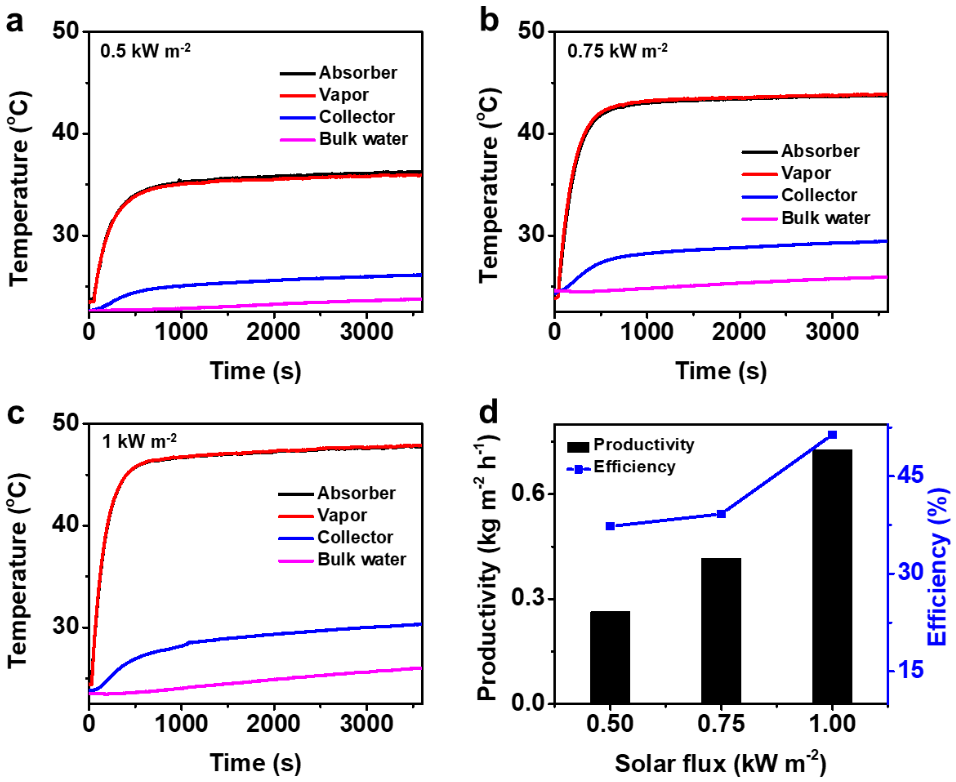

Figure S3. Water harvesting with one level collector device. The temperature of vapor and collector under (a) 0.5 kW m^{-2}, (b) 0.75 kW m^{-2} and (c) 1 kW m^{-2} solar flux. (d) The corresponding water productivity and solar efficiency under different solar

fluxes.
